# Improving the Promotion of Older Adults’ Physical Activity in Institutional Long-Term Care: Participatory Action Research

**DOI:** 10.1177/23333936261452490

**Published:** 2026-08-29

**Authors:** Noora Narsakka, Johanna Finskas, Riitta Suhonen, Barbara Groot, Jouko Katajisto, Minna Stolt

**Affiliations:** 1 University of Turku, Finland; 2 Turku University Hospital, and Wellbeing Services County of Southwest Finland, Finland; 3 Vrije Universiteit, Amsterdam, Netherlands; 4 Wellbeing Services County of Satakunta, Pori, Finland

**Keywords:** Action research, aged, environment, older adults, long-term care, nursing homes, nursing care, participatory research, physical activity, Finland

## Abstract

Older adults’ physical activity engagement in institutional long-term care is insufficient, despite the importance of physical activity for their health, functioning, and wellbeing. The purpose of this study was to improve the promotion of the physical activity of older adults in institutional long-term care by co-developing, implementing, and evaluating changes to care practices and the care environment in collaboration with staff members, older adults, and their family members. A participatory action research study was conducted in one unit with 18 staff members, 17 older adults, and 6 family members. Qualitative data were collected throughout the research process using interviews, focus groups, observations, workshops, and meetings, and analyzed with thematic analysis. Quantitative data collection was used to measure changes in older adult and staff outcomes before and after implementation of changes in activity promotion, and analyzed with statistical methods. Qualitative and quantitative findings were integrated in the results section. Three themes were developed, including (i) there is a need for a paradigm shift to improve physical activity promotion, (ii) change requires collaboration and time, and (iii) produced change can be beneficial in many ways and for different actors. Opportunities exist that are not currently used to promote older adults’ physical activity. Implementing physical activity in different parts of daily life and employing the environment could facilitate the sustainability of physical activity promotion. The role of nursing professionals is important in the implementation. Engaging staff members in co-development facilitates context-fitting change and care culture change, producing benefits also for themselves.

## Introduction

Physical activity plays a crucial role in maintaining the health, functioning, and wellbeing of older adults living in institutional long-term care settings. Due to advanced cognitive decline, significant mobility impairments, and high levels of care-dependency (Palese et al., 2016), most older adults living in a care setting need the support of other persons and the environment to engage in physical activity. Studies have consistently reported low levels of physical activity among this population (Hahn et al., 2023; Shi et al., 2025), suggesting insufficient promotion of this basic human need (Hahn et al., 2023) but also poor adherence to physical activity programs (Di Lorito et al., 2020). Although various interventions have been implemented to increase physical activity, many fail to address the complexity of factors related to older adults’ physical activity (Wylie et al., 2023), the influence of passivating care cultures, and the diverse needs of older adults (Bowes et al., 2022). To overcome the persistent issue of sedentarism in institutional long-term care, it is vital to involve both staff and older adults in generating insights concerning feasible and sustainable physical activity promotion. This article presents findings from a participatory action research project conducted together with staff, older adults, and their family members to improve older adults’ physical activity promotion by changes in the care and care environment within an institutional long-term care unit.

## Background

For health benefits, older adults in the institutional setting are recommended to engage in physical activity training at least twice a week, including 35 to 45 min of moderate-intensity (increasing heart and respiratory rate) endurance, muscle strength, balance, and coordination training adapted to each individual’s functioning (Peyrusqué et al., 2023). Physical activity is defined as “*movement produced by skeletal muscles resulting in energy expenditure*” (Caspersen et al., 1985). For older adults in the institutional long-term care setting, being physically active can help to maintain their physical performance, independence in activities of daily living, prevent falls and fractures, decrease the risk of depression, dementia and cancer (Cunningham et al., 2020), and slow down the progress of dementia (Paillard et al., 2024). Unfortunately, older adults in care homes spend most of their time being sedentary—that is, not moving (den Ouden et al., 2015; Parry et al., 2019)—and their needs for physical activity are often missed (Narsakka et al., 2023; Poveda-López et al., 2022). This has detrimental effects, possibly decreasing physical functioning (Ferrucci et al., 2016), leading to an increased risk of falling (Ho et al., 2021) and higher care dependency (den Ouden et al., 2015). Also, being sedentary is associated with falls, pressure sores, malnutrition, and urinary incontinence (Lahmann et al., 2015). Even increases in low-intensity activity, such as activities of daily living, may be beneficial (Mc Ardle et al., 2021), maintaining or improving physical functioning (Baldelli et al., 2021).

There is considerable potential to enhance older adults’ physical activity promotion in the institutional long-term care setting. Various promotion interventions have been tested, including, for example, exercise (Wylie et al., 2023) and promoting independence in activities of daily living (Hirt et al., 2024). Despite the availability of evidence-based interventions, a persistent gap remains between research and practice. Barriers to the sustainable delivery of physical activity include limited staff resources, low-motivated staff, and a failure to address the needs of older adults (Hirt et al., 2024; Quasdorf et al., 2023). Furthermore, in institutional long-term care, safety and resource efficiency are often prioritized over physical activity promotion, leading to restrictive policies and mechanistic approaches to providing care (Narsakka et al., 2022).

To overcome these challenges, scholars have emphasized the importance of addressing multiple interrelated factors, such as the physical and social environment, organizational factors, care culture, values, and policies (Anderiesen et al., 2014; Narsakka et al., 2022; van Alphen et al., 2016; Wylie et al., 2023). To achieve a simultaneous change in these factors, the engagement of staff and older adults is vital (Bowes et al., 2022). So far, few studies have explored improving physical activity promotion through participatory research approaches. These approaches provide opportunities to address various factors on different levels, simultaneously producing a change in the people, context and conditions (Feekery, 2024) to co-create a change for physical activity promotion. In nursing research, action research approaches have been used to improve professional practice (De Chesnay, 2015; Glasson et al., 2008), but are less common in institutional long-term care with older adults (Corrado et al., 2020).

To build on these insights, we conducted a participatory action research project with the purpose of improving the promotion of the physical activity of older adults in institutional long-term care by co-developing, implementing, and evaluating changes to care practices and the care environment in collaboration with staff members, older adults, and their family members. The study was guided by the research question: What insights can be gained about improving physical activity promotion in institutional long-term care through the process of co-developing, implementing, and evaluating changes to care practices and the care environment?

## Methods

### Design

We conducted the study by using a participatory action research design (Kemmis et al., 2014), using mostly qualitative data complemented with quantitative data. Participatory action research is rooted in praxis and critical theory (Kemmis et al., 2014, Lewin, 1946). The emphasis on data generation was qualitative, to conduct a collaborative inquiry, producing in-depth contextual understanding, and a transformative change within individuals, environments, and institutional practices (Feekery, 2024), through a cyclical process of learning by doing (O’Brien, 2001, pp. 2–3). We also generated quantitative data to enhance understanding of the impacts on both older adults and staff. This provided a robust understanding of the dimensions that could not be fully captured through only one of these methods (for more details, see chapter 3.5 Data collection). Theoretically, the study was founded on the conceptualizations of nursing, focusing on the dimensions of client, practice, and environment of the nursing metaparadigm (Kim, 2010), and understanding of the relationship of the older adult and the environment as depicted in the ecological model of environment and aging (Lawton, 1977, 1989).

One action research cycle was conducted in the study, including the phases *observe*, *plan*, and *act*, and *evaluate*. In the *observe* phase, a comprehensive assessment concerning the current state of the environment and care relating to the physical activity of older adults, and older adults’, family members’, and staff’s needs, preferences, and priorities for physical activity promotion in the unit was conducted. In the *plan* phase, based on the results of the previous phase, strategies to address the identified needs were co-created and selected to be implemented. In the *act* phase, planned strategies were implemented and their implementation was monitored. In the *evaluate* phase, the research process, its outputs and outcomes were evaluated and future needs and plans were reflected upon.

### Study Setting and Recruitment

This study was conducted in Finland. In Finland, institutional full-time long-term care is provided for older adults having extensive care or safety needs impeding living at home or in assisted living settings (Act on Supporting the Functional Capacity of the Older Population and on Social and Health Services for Older Persons 28.12.2012/980, 2012). At the end of 2023, 4% of persons aged 65 and over and 8% of persons aged 75 and over lived in institutional long-term care (Finnish Institute for Health and Welfare, 2024). Most older adults in these settings need care due to functional losses caused by cognitive decline, and 70% have a formal diagnosis of dementia (Edgren et al., 2024).

The study was conducted in a city in Southern Finland by a team of co-researchers, including academic co-researchers and practice-based co-researchers. We purposively recruited (Patton, 2015) an institutional long-term care unit interested in increasing the physical activity of older adults and developing their physical activity promotion practices. The participating unit was a home for 25 older adults, whom we refer to as older adults or residents. The unit was a part of a facility with several closed units, where the older adults could not exit independently. Older adults rented a room and used common areas in the unit, such as the dining area and living room. The facilities had additional shared spaces to organize events, a gym, a restaurant, a hair salon, and a fenced outdoor area. Staff working in the unit were mostly licensed practical nurses with a 3-year vocational education (180 ECTS). In addition, the staff included nursing aids without an official degree in social and health care, and registered nurses and rehabilitation professionals having a three and a half-year bachelor’s education (210 ECTS). The word *nurse* is used in the article to refer to practical nurses, nursing aids, and registered nurses.

All older adults living in the unit, their family members, and professionals working in the unit were eligible to act as practice-based co-researchers if they were capable of providing informed consent in the Finnish language. Recruitment was ongoing as new residents moving into the unit, their family members, and staff starting to work in the unit during the study period, were also asked to join. We did not ask for reasons to decline acting as a practice-based co-researcher. Altogether, 41 persons were included as practice-based co-researchers. They included staff members (*n* = 18), older adults (*n* = 17), and family members (*n* = 6). Their characteristics are presented in Table 1. Staff members were practical nurses (*n* = 9), registered nurses (*n* = 2), nursing assistants (*n* = 2), physiotherapists (*n* = 1), occupational therapists (*n* = 1), social workers (*n* = 1), and head nurses (*n* = 2). Family members were spouses (*n* = 5) and a child (*n* = 1) of the residents.

**Table 1.** Practice-Based Co-Researchers and Their Characteristics.

Characteristic	Older adults (*n* = 17)	Family members (*n* = 6)	Staff members (*n* = 18)
Women, %	59	33	94
Age, range	65–102	70–78	21–66
Age, mean	83	73	43
Walks independently with/-out aid, %	47.1		
Ambulates independently with wheelchair, %	11.8		
Walks with aid supported by nurse, %	17.6		
Ambulates passively with wheelchair, %	23.5		

### Shared Ownership and Management of the Research Process

A steering group of co-researchers was formed at the start of the study. The purpose was to facilitate shared ownership of the research project and enable collaborative decision-making, enhancing democracy and creating equal partnerships between the practice-based co-researchers and academic co-researchers (Tiefenthaler et al., 2022). The steering group included a staff member from all professional groups and the first author. We were unable to recruit older adults interested in and capable of collaborating in the steering group. Family members were invited, but there was no interest expressed. The steering group met a total of seven times to jointly plan the study process, and plan and organize data collection, the implementation of changes, and their evaluation. The first author prepared the meetings and facilitated discussion. In addition, the progress of the study was discussed in staff meetings (*n* = 6) of the unit to inform staff and facilitate discussions and decision-making. Discussions and decisions were recorded as notes, memos, or other materials, and used as qualitative data in the analysis.

### Data Collection

**Figure 1.**
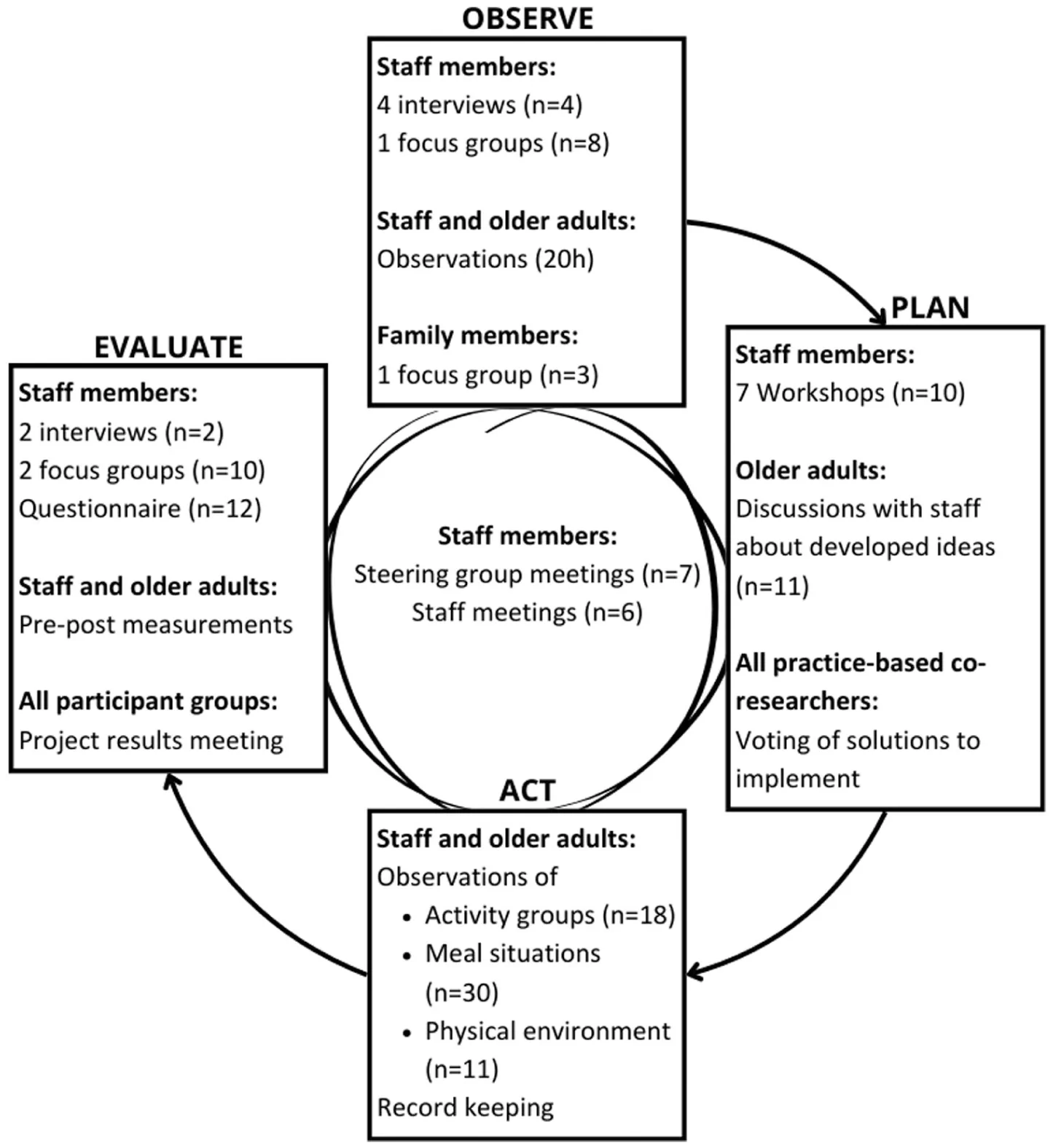
Overview of data collection in the cyclical research process.

Qualitative data were collected throughout the research process as practice-based co-researchers’ perspectives, experiences, and collaboration for change were the focus of the research (Dedding et al., 2022). The methods used in this research were selected together by co-researchers to facilitate the purpose of each phase and to involve all practice-based co-researchers in the process. This also allowed data crystallization, that is, integrating multiple perspectives to produce deep interpretive insights (Wilson, 2025). Quantitative data collection was used to measure changes in older adult and staff outcomes before and after implementation of the changes. An overview and exact numbers of the data collection are presented in Figure 1. The data were collected between March 2023 and June 2024.

#### Qualitative Data Collection

Focus groups and interviews were used with staff (*n* = 12) and family members (*n* = 3) in the observe and evaluate phases. NN collected these data. A homogenous approach was used as the practice-based co-researchers’ roles differed in relation to the topic and to allow their perspectives to be shared openly; family members and nurses participated in focus groups, and other professionals in individual interviews. Each session lasted between 1.5 and 2.5 hr and was recorded and transcribed verbatim by JF.

The interviews and focus groups were semi-structured (Patton, 2015). Guides were developed based on earlier research (Narsakka et al., 2022; Narsakka et al., 2023), and finalized together with the practice-based co-researchers in the steering group. In the observe phase, discussed topics included (1) daily life in the unit, (2) older adults’ functioning, (3) older adults’ weekly schedule, planning, and execution of physical activities, (4) actions and activities of personnel, family members and other people in relation to older adults’ physical activity, (5) the physical environment in relation to older adults’ physical activity, (6) opportunities, needs, and priorities for improving physical activity promotion, and (7) older adults’ individuality and involvement of family members in physical activity promotion. In the evaluate phase, the implemented strategies were evaluated for (I) what was working and why, (II) factors affecting implementation, (III) perspectives on the progress of implementation, (IV) achieved change during the study, (V) perceived impacts on older adults and staff, and (VI) producing change through conducted study activities and how the process could have been better. For evaluation purposes, staff members (*n* = 12) were also asked to anonymously comment on the research process and produced outcomes by using an online questionnaire developed for the purpose.

NN conducted observations throughout the research process and recorded them as field notes. With older adults, she used participatory observations (Patton, 2015) complemented with discussions and doing things together with them (Phillipson & Hammond, 2018). This approach was selected to include the perspective of older adults in the project, most of whom were experiencing cognitive decline. Observations were also used to observe the actions and activities of staff, and to record the change process. Furthermore, observations were an important means at the start of the study to build up rapport between the first author and the practice-based co-researchers.

Observations in the observe phase (20 hr) were conducted in 2 to 3-hr sessions between 8 am and 6 pm. In the act phase, to observe implemented changes, NN visited the unit unannounced during meal times (*n* = 30) and activity groups (*n* = 18), to observe whether the staff organized the agreed new activities and how. In addition, being present in the unit, she observed how the new physical environment was used and conducted activities with the older adults using the new features of the physical environment (*n* = 11). During observations, notes were taken on: place, people present or participating, the physical environment and objects, activities, purpose and timing of activities, and interactions, feelings, and reactions of people participating.

Workshops (*n* = 7) were conducted to ideate and co-design new solutions to promote physical activity with staff members (*n* = 10) in the phase plan. The content and structure of the workshops were developed by NN and JF. NN facilitated the workshops and JF made notes. We educated staff members with evidence on older adults’ physical activity and related factors (e.g., de Souto Barreto et al., 2016; Peyrusqué et al., 2023), and used creative methods (Groot & Abma, 2022) such as creating vision boards to facilitate ideation and discussion.

We organized a voting process to select solutions to implement in the plan phase. Materials produced in the workshops were presented as a gallery in a hallway of the unit, and all practice-based co-researchers were asked to give a maximum of five votes on their most preferred ideas to implement. Simultaneously, staff and family members were asked to provide additional comments regarding the ideas in writing. Staff members had individual discussions with older adults (*n* = 11) concerning the older adults’ preferences for physical activities, noting these down in writing and helped them to vote.

#### Quantitative Data Collection

To evaluate changes in older adults and staff outcomes, we collected quantitative data before and after the implementation of changes. The pre-measurements were conducted before the implementation of the first change in October 2023, and the post-measurements were conducted 16 weeks later. Implementation of the changes happened gradually, and, therefore, the changes had been in use for varying durations (5–12 weeks) before the post-measurements.

The primary outcome was older adults’ physical activity measured for seven-day periods by using the Fibion SENS Motion activity tracking system (Fibion Inc., 2020) worn on the thigh. The activity tracker is a triaxial accelerometer, registering the orientation and acceleration of the thigh. The registered data is categorized with the system’s algorithm into predefined activity categories, including: (1) light intensity activity (standing, sporadic walking, continuous walking), (2) moderate intensity activity (fast walking, cycling), and (3) high intensity activity (running, exercise). All activity segments were added together for weekly activity minutes. The same device on the back measured the time spent lying down. The system has been evaluated to be valid and reliable in measuring time spent in physical activity, sitting, and lying with older adults with poor mobility (Pedersen et al., 2022).

Secondary outcomes included life-space, quality of life, and person-centered care. Life-space relates to the extent of the space that older adults use when moving within the facility and how frequently they do so (Johnson et al., 2020). This was measured by using the Nursing Home Life-Space Diameter instrument (Tinetti & Ginter, 1990) by nurses keeping a record of older adults’ movement within the facilities for 7 days, and points were calculated based on these. Quality of life was measured by using the World Health Organization Quality of Life (WHOQOL -BREF) instrument (Goes et al., 2021). Person-centered care of the unit was evaluated with the Person-Centered Climate Questionnaire (Edvardsson et al., 2015) for residents and staff members before and after the implementation of changes. Questionnaires with older adults were conducted by the first author by using extra-large font, smiley faces corresponding to the answer options, and reading aloud/helping to fill in the forms to allow as many older adults as possible to participate.

Quantitative data was also collected concerning the implementation of changes as record keeping in the phase act, as staff noted down the organization of new activities, including information on the date of activity; nurses facilitating; older adults actively taking part, inactively taking part, and declining to participate; and the content of activity. Patient record transcripts regarding activities were collected for the same seven-day periods as the physical activity and life-space were measured to assess other simultaneous physical activities and changes in these.

### Data Analyses

In accordance with the research approach, data analyses in the cyclical process were ongoing and the results were fed to the process to facilitate knowledge-informed change (see Figure 1, Table 2). Furthermore, the data were used to answer the research question at the end of the process by merging qualitative and quantitative findings, using a narrative discussion in the results section (Creswell, 2017). Reflexive thematic analysis (Braun & Clarke, 2006; Braun & Clarke, 2022) was used to analyze qualitative data, conducted by using NVivo 1.7.1, Excel spreadsheets, and Word documents. The data were read and inductively coded by NN, formulating themes to answer the research question. A member check of the analysis was conducted with staff members in December 2024, and was used to refine the analysis. Quantitative data were analyzed by using descriptive statistics (frequencies, means, medians, ranges). Student’s *t*-tests were used to compare pre- and post- measurements. Normality of distribution was assessed by using histograms. Two-sided p-values were reported. Quantitative data analyses were conducted by using SPSS 29.0.0.0.

**Table 2.** Overview and Examples of Phase-Specific Outputs of the Study.

**OBSERVE**					
Six development themes formulated about practice-based co-researchers needs and wishes for increasing physical activity					
Physical recreation	Exercise	Daily chores	Outdoor activity and excursions	Physical environment	Social environment
**PLAN**					
Plethora of ideas and implementation strategies developed for the six development themes to increase physical activity (examples provided below)					
Games, baking, taking care of animals, mural painting	Gym exercise, chair exercises, dancing, stretching, balance trail	Being more active during meal times, taking part in laundry tasks, organizing one’s room	Excursions to nature, marketplaces, restaurants, sports events, cultural events, religious places	Scenery tapestry, plants, pictures of animals, resting places in hallways	Engaging family members, neighbors with animals, different organizations in organizing activities
Implementation strategies: • Implementing changes gradually, not all at once • Clear definitions for activities on what is done, when, how, and by whom • Rotating shifts for nurses to organize activities • Having equipment at close proximity and ready to use					
**ACT**					
Three changes implemented during the study					
Recreation and exercise: Afternoon group activity sessions		Daily chores: Promoting independence in meal situations		Physical environment: Activating hallways	
• A group by two nurses for 4–6 residents • Daily in the afternoon • 15 min exercise and 15 min of other activities • Ready-made plans to organize groups (*n* = 13) by nurses with varying content • All necessary equipment available in a cart		• Drinks and bread placed on tables to allow residents to serve themselves, guided by the nurses • Guiding residents to the hot plate in the kitchen to take part in serving their own hot meals • New meal time roles for nurses to facilitate activating older adults		• Ten sections of activating walls created in two hallways ○Different physical exercises with equipment ○Pictorial, mathematical, verbal, and functional activities • Decorated resting places • Chairs placed along the hallways	
**EVALUATE**					
Research process, its outputs and outcomes evaluated and future needs and plans reflected					
• Perceived increase of physical activity and activities • No positive statistically significant changes between pre- and post-measurements • Continuing implementation of strategies after study period • Need to involve older adults and family members more in the future					

### Ethical Considerations

We conducted the study according to the ethical guidelines of the Finnish National Board on Research Integrity (TENK, 2019, 2023), the European Federation of Academies of Sciences and Humanities (ALLEA, 2023), and the ethical principles of participatory health research (International Collaboration of Participatory Health Research, 2022). Before the start of the study, we obtained ethical approval, later making two amendments, from the Ethics Committee for Human Sciences at the University of Turku, Health Care Division (TY/40/12.12.2022, TY/699/06.01.01/2023, TY/131/06.01.01/2024) and obtained a research permit (2023-001224 T 13 02 01) from the collaborating organization.

Academic co-researchers provided information letters in the unit to be distributed to practice-based co-researchers. Before collecting consents, all practice-based co-researchers were informed orally and in writing about the purpose of the study, the research process, possible harms and benefits, voluntary participation, and the rights of practice-based co-researchers, and were provided with the opportunity to ask questions. Older adults’ capability to provide informed consent was assessed by staff members who knew the older adults well and made similar assessments for care provision. When incapable, a family member was informed and provided informed consent on behalf of the older adult. The personal data of all practice-based co-researchers were pseudonymized or anonymized in accordance with Finnish (Data Protection Act 1050/2018) and EU legislation (General Data Protection Regulation, Articles 13 and 14, 2016). Influence on persons living and working in the unit, not participating in the study, was carefully considered during the research process, especially regarding the vulnerability of older adults.

### Rigor and Reflexivity

The academic research team was comprised of white people, mostly women, living in European countries. The first author is an early career researcher having clinical work experience working with older adults in the long-term care setting. The second author worked as a research assistant in the study, working simultaneously in the collaborating unit 1 day per week as an occupational therapist and conducting master’s degree studies in nursing science. The other researchers have extensive experience in research in the field of older adults’ health, care, and rehabilitation, or statistical methods. BG is an expert in participatory research with older adults.

To ensure the rigor of the study, we have reported our theoretical stance, descriptions of the study context and practice-based co-researchers, positionalities of the academic co-researchers, and the process and outcomes of the study. Consistent with our theoretical stance, decisions within the study were made and evaluations of the process and its outcomes were conducted together with the practice-based co-researchers. For these purposes we used various data collection methods and conducted iterations within the process, increasing the credibility of the study (Lennie, 2006). An audit trail was recorded by using various materials collected and produced within the process. Prolonged engagement was fundamental to the research process, and NN used reflexive diaries for reflexive practice (Walsh, 2003) in the field.

## Findings

Working through the process of co-developing, implementing, and evaluating changes to care practices and the care environment, we produced various phase-specific outputs (Table 2): We formulated six development themes of the practice-based co-researchers' needs and wishes for improving physical activity promotion (observe phase). These included physical recreation, exercise, daily chores, outdoor activity and excursions, physical environment, and social environment. Co-designing solutions to meet these needs and wishes (plan phase), various ideas for new activities and opportunities for activity promotion were developed, varying from simple activities incorporated into daily life to organizing events. Also, strategies for their implementation were created. In the voting by older adults, family members, and staff, altogether 94 votes were given. Of the most voted for ideas, three strategies were composed to be implemented in practice by focusing on different identified needs. The strategies included organizing afternoon group activity sessions, promoting independence in meal situations, and employing hallways in activating older adults. Implementing these strategies gradually (act phase), meal time changes were implemented for 16 weeks, activity groups for 13 weeks, and activating hallways for 1 week between pre- and post-measurements of outcomes. The implementation was supported by ongoing discussions and by responding to emerging needs, for example, by improving how agreed changes were communicated to all staff and by agreeing to remain flexible when unexpected situations arose. Evaluating the research process (evaluate phase), the three strategies were perceived to have increased older adults’ opportunities for physical activities but also to have produced various other benefits for older adults and staff, despite no positive changes being observed in the pre- and post-measurement of outcomes.

To generate insights about improving physical activity promotion through this process, we developed three themes in the analysis at the end of the study. These include: (1) *there is a need for a paradigm shift to improve physical activity promotion*, (2) *change requires collaboration and time*, and (3) produced *change can be beneficial in many ways and for different actors*.

### There is a Need for a Paradigm Shift to Improve Physical Activity Promotion

Based on the qualitative data collected in the observe, plan, act, and evaluate phases, there seemed to be a large discrepancy between the existing reality and the perceived possible reality of older adults’ physical activity in institutional long-term care. We defined this to reflect a need for a paradigm shift in physical activity promotion. Paradigm here is defined as a set of shared assumptions and beliefs about reality that guide people’s interpretations and actions (Schein, 2017, pp. 34–38).

The reality was that older adults did not engage in much physical activity and did not have many opportunities for physical activities. Their life included mostly basic activities of daily living, ambulating within the unit if capable of doing so, and infrequent outdoor visits and passive activities.

I ask her [resident of the unit] how it was going. She replies, “uneventful” and soon continues,”the same thoughts go around in my head . . . would you live in an environment like this?” . . . I ask her what she thinks about the environment. “Well, like this, that nothing happens.” (observation note)

Passivity was perceived as normal. For example, family members could not really grasp what their loved one could even physically do.

I cannot think of anything, when they are just sitting and not taking any part in the world, not taking part in any events, so it’s quite difficult to say what you could then develop [to increase their physical activity]. (family member)

Most older adults needed sustained guidance, physical support, or both to actively take part in physical activities. Promoting physical activity required considering their cognitive and physical functioning, sensory condition, illnesses, medications, the capacity to focus, varying energy levels, and mood. A staff member told, “*We have a lot of [residents with] memory disorders. It depends; in the morning, they can be very active, but in the evening, it can be difficult. . . . then they need more help from the nurses.*”

However, if and when receiving sufficient physical activity promotion, it was perceived by staff that older adults could be more physically active at a higher volume, intensity, and more frequently than they presently were. In the perceived possible reality of a more physically active daily life, older adults would have the opportunities for and would engage in different activities regarding physical recreation, exercise, daily chores, outdoor activities, and excursions. Also, the physical and social environment would be used in different ways to promote physical activity, which was not the case in the current paradigm.

So if there would be like some objects or stuff, that would somehow motivate to move forward, or to go see what’s there or. And they, in their part, also make it homier and break like this kind of hallway feeling. (staff member)

In bridging the gap between the existing reality and the possible reality, the staff members benefited from education about the importance of physical activity and ways to improve it, opportunities to critique their own beliefs and assumptions about older adults’ physical activity and abilities, and opportunities to develop their own practices and the environment. A staff member described that “*through the study discussions, co-designing, and evaluation, I have become more aware about the opinions people in our unit have regarding older adults and their physical activity.*” To produce change in physical activity promotion, the staff perceived that especially collaboration and time were vital. This is discussed in the following theme.

### Change Requires Collaboration and Time

Throughout the research process of observing, planning, acting, and evaluating, staff members shared their thoughts and had discussions on what would be required for successfully producing improvements in physical activity promotion. Two things—collaboration and time—were considered of special importance.

To improve physical activity promotion, having open discussions and ideating and co-designing the changes together between different professionals was seen by staff as integral to achieving long-lasting solutions in everyday practice.

This job includes specific things . . . it is a part of it that we are with the residents and we help them with everything they need our help with. But the fact that you can produce [change] through positive means, that is what makes change possible . . . it is always better that people figure out things themselves. . . . They are the experts in their own jobs, and if they get to be a part of developing and planning it, how things are done, when they are done, how they could be executed, that could improve physical activity promotion. (staff member)

All professionals in the unit participated in the study activities, but especially the involvement of nurses was perceived as important, as they were the ones implementing the co-designed changes. Their feeling that older adults’ new activities would be unmotivating extra work was perceived as a possible barrier to achieving change. By having an active role in the process, their interests could be employed to increase their motivation toward physical activity promotion.

The staff themselves were able to be involved in creating a new active daily life from the point of view of our residents. It is important for the sustainability of the changes for them to be accepted by the staff. (staff member)

Nurses’ engagement in the process also enabled the production of changes that would be feasible in their everyday practice. For implementing change, the frequency, duration, and moment in time for the chosen changes were important considerations. The fact that staff members were able to plan these things and have ongoing discussions about them during the implementation and evaluation phases facilitated success in improving physical activity promotion. A head nurse told that her job in the process was “*to take care that we follow the situation [of implementation] and reflect where we go and have discussions about that. And then inform [everyone] about what we have decided*.”

Even though collaboration was perceived as a key component for producing change, this was somewhat limited to collaboration between professionals. Understanding and employing older adults’ motivation, personal interests, preferences, and life history for physical activity were identified as important for physical activity promotion. However, these factors were not largely focused on in the delivery of the new strategies. The co-produced changes were perceived to offer “something for everyone,” but in practice, older adults’ interest and engagement in the changes varied.

At the end of the project, staff perceived that older adults could have been more involved in the process of co-producing changes to better meet their wishes, as the staff now had the leading role. Advanced cognitive decline was perceived as a barrier for higher involvement, but could have been tackled with strategies such as employing staff and family members' knowledge of the older adults’ preferences and history, and having more research activities focused on the individual preferences of the older adults. At the end of the research process, it was proposed that future development goals could include better consideration of older adults’ preferences, but also involve family members in physical activity promotion.

All in all, progressing little by little in improving physical activity promotion was perceived as a necessity. Learning out of old routines and doing things in new ways was considered to be hard. Therefore, change should happen slowly and progressively so that previous changes were internalized before new ones were implemented. Also, those staff members who were more interested and motivated for the change from the start would act as exemplars and motivate others. With this, change would spread throughout the organization little by little.

### Change Can Be Beneficial in Many Ways and for Different Actors

This theme was developed using qualitative and quantitative data from the act and evaluate phases to represent the perceived benefits of co-developing, implementing, and evaluating changes in care practices and the care environment. We did not observe statistically significant positive changes in older adult outcomes, but their opportunities for physical activities were perceived to have improved. In addition, staff perceived that the research process had produced various other benefits for the older adults, but also for themselves.

Considering the main outcome of the study, physical activity, staff members considered that older adults’ physical activity and opportunities for activities had increased due to the implemented changes of organizing afternoon activity sessions, activating older adults during meal times, and employing hallways to activate older adults. Older adults had more recreational activities and exercise due to the afternoon group and were more active during meal times. One staff member said, “*These afternoon groups have considerably increased this kind of activity. Even if it’s not executed one hundred percent. . . . I think it has increased activity. . .*” During observations, older adults were observed to enjoy the new activities.

Several people seemed to enjoy the ball game. One resident spontaneously said, “Good that you have invented this.” He/she was laughing and smiling during the game and was in a good mood and actively threw or pushed the ball when it came to him/her. (observation note)

**Figure 2.**
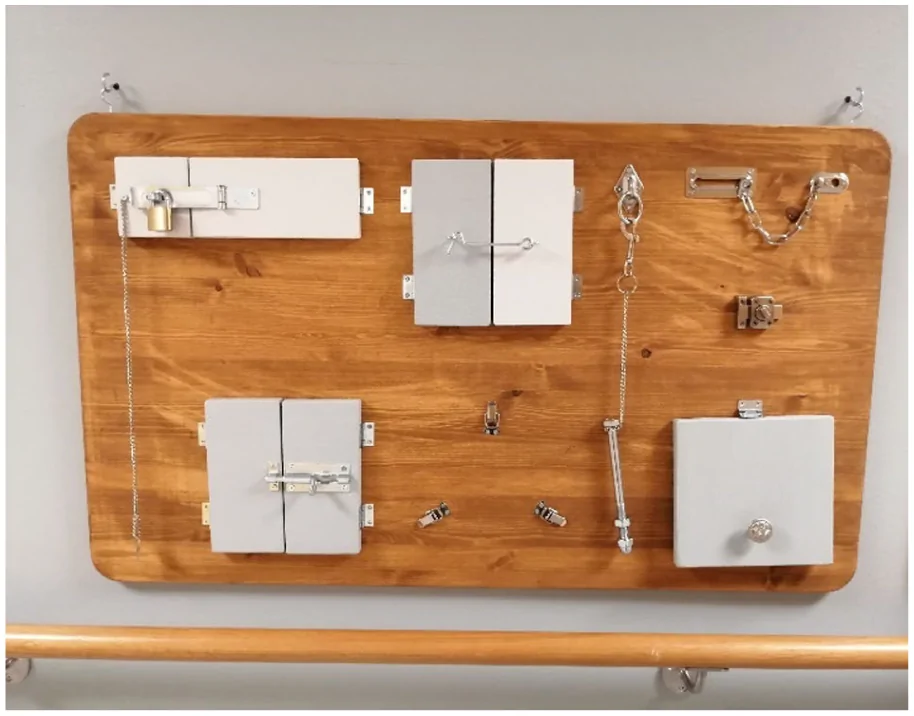
Wooden panel (Photo: NN).

**Figure 3.**
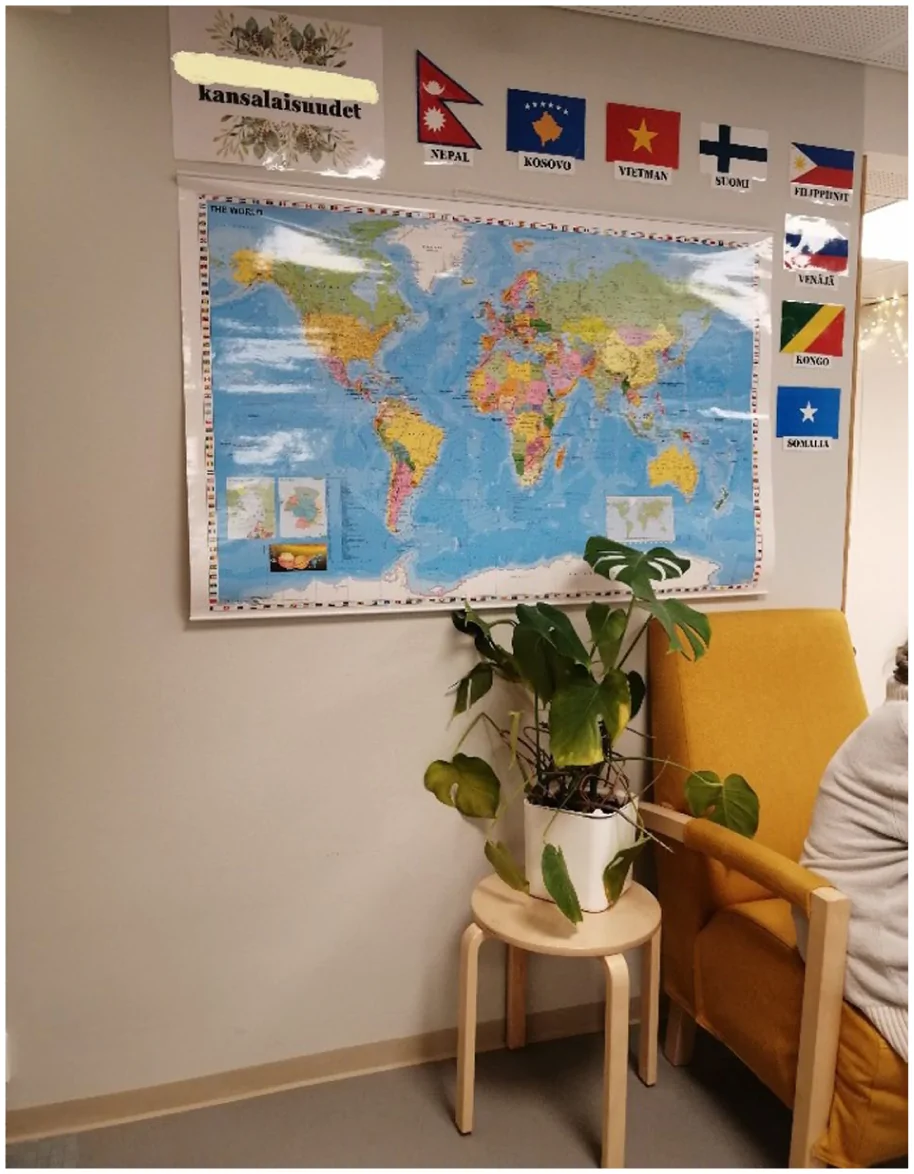
Resting place (Photo: NN).

There were more opportunities for stimulating physical activities with the physical environment (Figures 2 and 3); however, most older adults were dependent on the initiation and guidance of nurses or family members, and only a few benefited from the physical environment on their own. The resting places were frequently used by independently ambulating older adults.

The results of statistical analyses on resident outcomes are presented in Table 3. A statistically non-significant rise in older adults’ physical activity was observed in actigraphy measurements, as the physical activity of older adults (*n* = 12) was on average 7.72 hr (463.34 min)/week during pre-measurements and 9.05 hr (542.81 min)/week during post-measurements (*p* = .399).

**Table 3.** Results of Statistical Analyses on Resident Outcomes.

Outcome	*N*	Test	Min	Max	Mean	*SD*	*t*	*p*
Physical activity, min/week	12	Pre-test	1.11	1,623.76	463.34	504.12	−0.878	.399
		Post-test	1.11	1,516.78	542.81	549.99		
Lying down, min/week	13	Pre-test	4,665.33	8,292.35	6,770.29	879.27	−1.778	.101
		Post-test	4,778.69	7,708.20	6,543.89	901.57		
Life-space (NHLDS)	13	Pre-test	10.00	33.00	23.08	7.65	−2.667	**.021** *
		Post-test	12.00	29.00	18.46	5.41		
Quality-of-life (WHO-QoL-Bref)	7	Pre-test	238.00	350.00	266.71	38.44	−0.845	.431
		Post-test	151.00	325.00	250.57	65.66		
Person-centred care (PCQ-P)	5	Pre-test	52.00	81.00	70.40	11.55	−9.431	<.001*
		Post-test	40.00	69.00	57.40	11.52		

*Note*. NHLSD = Nursing home life-space diameter (Tinetti & Ginter, 1990). WHO-QoL-Bref = World Health Organization quality-of-life short version (Goes et al., 2021). PCQ-P = Person-centered climate, patient version (Edvardsson et al., 2015).

* *p*-value ≤ .05 are bolded.

On the contrary, life-space (*n* = 13) statistically significantly decreased between pre- (mean 23.08 points) and post-measurements (mean 18.46 points; *p* = .021). This was mostly due to a decrease in outdoor activity between the pre-measurements conducted in the fall and the post-measurements conducted in the winter. Also, in the quantitative measurements, a negative outcome for older adults was observed as older adults’ (*n* = 5) person-centered care statistically significantly decreased between pre- (mean 70 points) and post-measurements (mean 57 points; *p* < .001). Other changes between older adults’ pre- and post-measurements were not statistically significant.

Staff members perceived that various benefits in addition to physical activity promotion improvements were produced. Residents made more choices, had better food and drink consumption, and socialized more with other residents and staff due to the changes. A staff member described that “*many eat then the whole plateful better, when they see, when they get to say how many potatoes they take. Even if they don’t take it themselves, but when they see that it’s put there [on the plate]*.” The changes also benefited those who were no longer capable of physical activity. For example, in the afternoon groups, older adults incapable of active participation could still come and enjoy the activities and others’ company passively.

According to staff, various benefits were produced for themselves as well. Staff members considered that the change in meal situations increased their collaboration, and work was divided more equally. Meal times were calmer, which benefited residents and nurses, and residents’ independent physical activity could relieve nurses’ workload. The afternoon groups were considered to bring joy to the nurses in addition to the residents. The nurses learned new things about physical activity promotion, but also about residents’ functioning as residents engaged in new activities. Overall, the co-creation process was perceived to positively affect the workplace atmosphere. As a downside, the nurses said that the new activity group added to their workload and sometimes created stress if the day’s schedule was busy. No significant change was observed in the staff members’ (*n* = 10) person-centered care climate points between pre- (mean 60 points) and post-measurements (mean 58 points; *p* = .459).

## Discussion

This study generated insights about improving physical activity promotion in institutional long-term care through the process of co-developing, implementing, and evaluating changes to care practices and the care environment. Our findings emphasize the need for a paradigm shift from that of enforcing passivity to one of promoting physical activity. A key finding of our study was that change without additional staff resources was possible, giving staff members an opportunity to learn about and become more aware of physical activity, develop their own practices, and the environment.

Based on our findings, there was a large discrepancy between the existing reality and the perceived possible reality of older adults’ physical activity and its promotion. Similarly to our study, older adults have been observed in other studies to mostly engage in indoor mobility and activities of daily living (den Ouden et al., 2015; Hahn et al., 2023). However, various opportunities for activities were identified in the present study, so much so that only a minority of them could be implemented. This highlights the vital need to develop physical activity promotion. Furthermore, the variety of opportunities emphasizes the importance of understanding older adults’ physical activity as a part of life throughout the day, in many forms and activities, and happening in relation to the surrounding environment. To increase older adults’ currently low physical activity levels (Hahn et al., 2023; Shi et al., 2025), approaches aiming to increase physical activity throughout the day could be more sustainable, instead of focusing on one kind of physical activity, which is a common approach in current literature (Wylie et al., 2023). For example, exercise is an often-studied approach to increase older adults’ physical activity, and has been found effective in producing various benefits for older adults in institutional long-term care (Peyrusqué et al., 2023). However, sustained implementation is often hampered due to delivery by physiotherapists or other external professionals (Wylie et al., 2023), whose resources are also scarce in the Finnish care context (Kehusmaa & Alastalo, 2021). Nurses as professionals spend most time with older adults, and could therefore engage them in various forms of physical activity throughout the day. Also, considering the importance of physical activity for an individual to take part in life activities and social interactions, comprehensive nursing care is vital. It should be noted that promoting independence in basic activities of daily living, which can increase older adults’ physical activity (Hirt et al., 2024), was not the focus of this study. This was due to the fact that nurses were perceived to already promote older adults’ physical activity in basic activities of daily living, which is reported in another article (Narsakka et al., 2025).

The social and physical environment was identified as a resource for physical activity promotion, not largely used so far. Regarding the social environment, various ideas were produced to improve physical activity promotion by engaging family members, volunteers, and the wider community. As institutional long-term care units are faced with a shortage of staff and nurse retention (Rostgaard et al., 2022), these groups could provide much-needed additional resources for physical activity promotion (Hahn et al., 2024). However, this study took a more professional-led approach because practice-based co-researchers chose to implement strategies other than social engagement. By the end of the study, they identified greater family involvement as a key area for future development. This could produce a better understanding of the personal preferences and life history of older adults living with dementia. In addition, the involvement of the social environment could help to tackle the prevalent problems of social exclusion (Paananen et al., 2024) and the loneliness of older adults in institutional long-term care (Edgren et al., 2024). However, we argue that all older adults should be entitled to engage in preferred activities, regardless of the involvement of the community, emphasizing the role of care professionals in meeting these needs.

Regarding the physical environment, accessibility in the institutional long-term care setting has been improved in the past decades, enabling mobility and physical activity (Narsakka et al., 2023; Narsakka et al., 2025). However, as depicted in the ecological model of environment and aging (Lawton, 1977, 1989), a suitable environment also produces sufficient stimulation meeting the abilities of the older adults. Based on our findings, different stimulating solutions could be produced in the environment with low costs that meet the needs of individuals with varying functioning levels. These can also create a more homelike environment, which may have additional benefits (Fleming et al., 2020). Furthermore, stimulating elements in the immediate environment provide opportunities for care professionals to organize physical activities without looking for equipment or inventing what to do. This is important, considering the strict time schedules of nurses (Hirt et al., 2024). Activating elements in the physical environment also provide opportunities for family members and other people to engage older adults in physical activities (Narsakka et al., 2023). In the present study, staff members co-created the implemented modifications to the physical environment, and in future research, the physical environment should be further explored with older adults themselves.

In the present study, the promotion of physical activity was improved by delivering new physical activities in different parts of daily life and using the physical environment. Despite improvements being achieved from the points of view of the practice-based co-researchers, no statistically significant improvement was perceived in older adults’ physical activity levels between pre- and post-measurements. Sample sizes in participatory action research are small (Zuber-Skerritt & Wood, 2019), which could have affected the non-significant result. Given that older adults typically experience functional decline over time, even a statistically significant stabilization or slight increase in physical activity could be encouraging. It should be recognized that the expected increase in the levels of older adults’ physical activity remained somewhat limited as well; only six out of 25 older adults per day participated in the activity group consisting of 15 to 30 min of physical activity, the implemented changes in meal times did not produce high increases in physical activity, and changes to the physical environment were introduced only 1 week before the post-measurements and were not yet fully in use at that time. Furthermore, not all changes interested or pleased all older adults, affecting the extent to which they engaged in the new physical activities. The varying motivations and interests of individuals can be addressed by using multiple strategies and physical activities planned with the older adults themselves (Maurer et al., 2019; Poveda-López et al., 2022), which was identified as a possible future development goal in the unit for improving physical activity promotion. With multiple action research cycles, which is typical in action research, we possibly could have addressed these and other needs identified during the process. However, only one cycle was conducted in the present study.

Notably, we observed decreases in older adults’ life-space and person-centered care climate between pre- and post-measurements. The reduction in life-space may reflect seasonal change, as pre-measurements were conducted in the fall and post-measurements in the winter. Winter-time outdoor activity in the Finnish care context is often lower than during other seasons, due to the harsh weather conditions and the increased practical effort of going outdoors (Narsakka et al., 2023). Wintertime outdoor activity was identified as a potential development area in the observe phase but was not selected to be improved, which may explain the decrease in life-space. For perceived person-centered care climate, interpretation should be cautious as the paired sample remained very small (*n* = 5). The observed change may reflect changes in the participants’ health and wellbeing status, influencing their perceptions of the care environment. However, as person-centeredness remained beyond the scope of this study, the result could have also been affected by the unmet individual needs and preferences related to physical activity promotion.

Rather than producing evidence on effective interventions, the contribution of this study to the literature is the success of improving physical activity promotion as nursing practice without additional staff resources. Common impediments for physical activity promotion by nurses in the institutional long-term care setting are not perceiving physical activity promotion as a part of the nurse’s role (Baert et al., 2016; Hallam & Lewis, 2022), and a lack of time and motivation (Hirt et al., 2024). In the present study, nurses’ role was developed to take more charge of physical activity promotion, and the keys in this were the collaboration of staff, allocating time to the research to co-design changes, and learning to use time in a new way to promote physical activity. This kind of approach also provides opportunities for care culture change (Etherton-Beer et al., 2021), which could be “the one big lock” that should be opened for changing the current paradigm of passivity into one of activity.

Furthermore, development of care and the environment should be evidence-based. This requires structure and tools available to staff (Forster et al., 2021), for which future research should contribute. In the present study, the existing care culture and environment were collaboratively challenged (Bowes et al., 2022) using evidence to increase staff members’ knowledge on the topic (Hirt et al., 2024; Salma & Waelli, 2023) and influencing, for example, professionals’ and the organizations’ perceptions about the capacity of older adults and their need and wishes for physical activity. Informing future actions with previous findings in the research process (Tracy, 2019) facilitated producing evidence-based but context-specific changes that the practice-based co-researchers had ownership of (Feekery, 2024), and the skills development of the staff co-researchers (Quasdorf et al., 2023; Salma & Waelli, 2023). To achieve permanent changes, 1 year is a short time (Underwood et al., 2013). However, small projects that meet the needs of stakeholders are important to ignite care culture change processes (Snoeren et al., 2016) that can lead to permanent change.

### Strengths and Limitations of the Work

A strength of our study was the collaborative, cyclical research process employed to meet the resources of the collaborating unit and the practice-based co-researchers. Older adults are often not involved as equal partners in participatory action research (Corrado et al., 2020), which is especially difficult in the institutional setting, considering older adults’ advanced functional decline. A strength of our study is that we succeeded in including them in the research process to identify things to change, vote on the things to implement, evaluate the implemented changes, and, in the outcome evaluation, even though staff had the most active role in the process. Due to the functional decline of older adults, the sample sizes for some measurements remained very low, despite efforts to increase participation by using visual aids and personal support to answer questionnaires. Other researchers have faced similar difficulties (Kruse et al., 2021).

Building relationships and trust was an essential part of our research. The first author spent a total of 180 hr in the field, becoming acquainted with the staff, the older adults, and their family members. Furthermore, these factors were facilitated by the second author working at the unit and for the study. The process relied on shared decision-making (Godfrey-Faussett, 2022), which was facilitated by an ongoing dialogue between co-researchers (Kemmis et al., 2014) and more formal structures, such as the steering group. We were able to achieve ethical symmetry—that is, symmetrical relationships between academic co-researchers and practice-based co-researchers (Tiefenthaler et al., 2022). Unfortunately, the collaboration of family members remained scarce. We were able to involve only a few family members, and only some of them collaborated in the study activities. In the co-creation phase, approaches to include family members more were also ideated, but these were not selected to be implemented. More focus should be given to including family members in future research.

Only one action research cycle was conducted in the study. This limited addressing the identified needs and optimizing emergent barriers and facilitators. Our original plan was to initiate the study earlier and conduct it for a longer period of time. However, due to the COVID-19 pandemic, we decided to postpone field work, which limited the timeframe to conduct this study within a larger research project. A second cycle most likely would have improved the implementation of the selected strategies and made it possible to implement also other ones.

The thigh-worn activity trackers were highly practical and user-friendly for continuous activity measurements. Other activity trackers may be removed by persons living with dementia, as they do not remember their purpose (Kukkohovi et al., 2024). However, the trackers measured physical activity only when standing up or cycling. Physical activity done while sitting, such as moving with a wheelchair and chair exercise, was not recorded and could have affected the measurement of a statistically significant change. Therefore, another activity tracker could have been a better choice to observe changes between pre- and post-measurements.

We were able to produce knowledge that can be used in other settings (International Collaboration of Participatory Health Research, n.d.) and, to do so, provided information to understand the conditions in which the knowledge was produced. As participatory research is a process characterized by messiness (International Collaboration of Participatory Health Research, n.d.), despite our best efforts to describe the conducted research based on the collected and produced data, the present article remains, to some extent, a simplification of the process.

### Recommendations for Research, Policy, and Practice

We recommend future research to develop structured, adaptable, evidence-based approaches for collaboratively improving physical activity promotion by the involvement of stakeholders and test these with larger samples, in multiple settings, for the long term, for example, as cluster randomized trials. Testing should be done involving older adults with advanced functional decline to represent the population living in these settings, which is often not the case in physical activity research (Wylie et al., 2023). Different solutions for the physical environment to activate older adults should be further explored together with older adults themselves to account for the heterogeneity of older adults, and their needs and wishes for physical activity in institutional long-term care.

Nurses’ roles should be clarified, and the physical activity care culture should be improved to integrate physical activity promotion by using various strategies as a part of everyday life. This is vital for improved practice. Nursing education should include more knowledge and skills development about the importance and practice of physical activity promotion. Furthermore, interprofessional collaboration is important for high-quality care (Doornebosch et al., 2022), and employing the competence of different professionals, such as physiotherapists and occupational therapists, in developing physical activity promotion practice could be used more in institutional care settings.

## Conclusion

Our study generated insights about improving physical activity promotion in institutional long-term care through the process of co-developing, implementing, and evaluating changes to care practices and the care environment. They have pointed to the need to shift from a culture of enforced passivity to one that promotes physical activity, the multitude of physical activities that older adults could engage in but are not currently supported to do so by their carers, and the opportunities for promoting their physical activity by employing the environment. Implementing activity in different parts of daily life could provide a more sustainable approach to physical activity promotion compared to single-component interventions. The role of nurses should be developed to facilitate older adults’ physical activity throughout daily life. Engaging staff members in the development of care practices and the care environment facilitates producing sustainable, context-fitting solutions and fosters care culture change. Older adults’ needs and preferences for physical activity are important to understand, to support them in engaging in physical activity. The findings were generated in a cyclical process of generating change together with staff, older adults, and family members within an institutional long-term care unit, and can be used to inform practice, policy, and research.
